# Integrative Proteomics and Tissue Microarray Profiling Indicate the Association between Overexpressed Serum Proteins and Non-Small Cell Lung Cancer

**DOI:** 10.1371/journal.pone.0051748

**Published:** 2012-12-19

**Authors:** Yansheng Liu, Xiaoyang Luo, Haichuan Hu, Rui Wang, Yihua Sun, Rong Zeng, Haiquan Chen

**Affiliations:** 1 Key Laboratory of Systems Biology, Institute of Biochemistry and Cell Biology, Shanghai Institutes for Biological Science, Chinese Academy of Sciences, Shanghai, China; 2 Department of Thoracic Surgery, Fudan University Shanghai Cancer Center, Department of Oncology, Shanghai Medical College, Fudan University, Shanghai, China; University Magna Graecia, Italy

## Abstract

Lung cancer is the leading cause of cancer deaths worldwide. Clinically, the treatment of non-small cell lung cancer (NSCLC) can be improved by the early detection and risk screening among population. To meet this need, here we describe the application of extensive peptide level fractionation coupled with label free quantitative proteomics for the discovery of potential serum biomarkers for lung cancer, and the usage of Tissue microarray analysis (TMA) and Multiple reaction monitoring (MRM) assays for the following up validations in the verification phase. Using these state-of-art, currently available clinical proteomic approaches, in the discovery phase we confidently identified 647 serum proteins, and 101 proteins showed a statistically significant association with NSCLC in our 18 discovery samples. This serum proteomic dataset allowed us to discern the differential patterns and abnormal biological processes in the lung cancer blood. Of these proteins, Alpha-1B-glycoprotein (A1BG) and Leucine-rich alpha-2-glycoprotein (LRG1), two plasma glycoproteins with previously unknown function were selected as examples for which TMA and MRM verification were performed in a large sample set consisting about 100 patients. We revealed that A1BG and LRG1 were overexpressed in both the blood level and tumor sections, which can be referred to separate lung cancer patients from healthy cases.

## Introduction

Lung cancer is the most frequent cancer in the world, in terms of both incidence and mortality. Non-small cell lung cancer (NSCLC) accounts for 80–85% of lung cancer with an overall 5-year survival rate less than 14% [1]. Specifically, the 5-year survival rate is barely 3% to 7% for stage IIIB, and is less than 1% for stage IV disease [2]. However, patients diagnosed at an early stage and have surgery experience an 86% overall 5-year survival [3]. Therefore, new diagnostics are needed to detect early stage lung cancer because it may be cured with surgery. Several potential protein signatures such as carcinoembryonic antigen, CYFRA21-1, plasma kallikrein B1 and neuron-specific enolase have been discovered and clinically used as biomarker candidates for lung cancer. Nevertheless, none of them showed enough sensitivity, specificity or reproducibility [4]. Hence, biomarkers for early diagnosis of lung cancer are urgently needed.

Molecular biomarkers for early detection of lung cancer can take many forms. Histological biomarkers are paramount because they can be directly associated with the pathological changes, the risk of contraction, the presence or the stage of disease. They are believed to have potential to distinguish the different molecular disease mechanisms of NSCLC. On the other hand, serum biomarkers for lung cancer are even more appealing, because blood is readily accessible and is thought to acquire proteins secreted, shed, or otherwise released from the tissues through which blood circulates. Even some moderate abundant plasma proteins could be indicators of special body status and have been reported to fluctuate in response to certain types of diseases [5].

Currently, the disease-driven proteomics based on mass spectrometry has been introduced to the discovery of both histological and serological biomarkers. Despite the importance of serum biomarker discovery, one of the major technical challenges has been the fact that blood proteome is extremely complex, spanning a concentration range of at least ten orders of magnitude. It is anticipated that efficient depletion methods and multi-dimensional fractionation systems might be helpful to separate low abundance proteins and extend of the detection limit [6]. Herein, we used an extensive fractionation on peptide level to profile the albumin depleted serum proteome, by a unique integrated multidimensional liquid chromatography (IMDL) system developed in our lab [7]. Another technique hurdle is how to quickly and efficiently compare protein levels across tissues or plasma samples. Usually, these samples are not compatible with in vivo stable isotopic labeling strategy of MS-based quantification. Sequentially, in vitro labeling strategies such as iTRAQ [8] and acrylamide isotopes [9] are emerging as alternatives. Nevertheless, these techniques have limitations associated with cost, usually smaller proteome coverage due to labeling selectivity, applicability and differences in labeling efficiency. In this study, we therefore utilized a simple and robust label-free quantification (LFQ) strategy by spectral counting in the discovery phase. Moreover, the pressing need for reproducible MS analysis has led to the development of multiple reaction monitoring (MRM) technique. This technique can be used to measure the protein concentrations in clinical plasma samples when integrated with synthesized peptide standards and absolute quantification analysis (AQUA). Better selectivity, sensitivity and dynamic range can be achieved by MRM, which are crucial for clinical validation [10].

The aim of this study is firstly to use shotgun proteomics to directly discern the differential serum proteome patterns associated with NSCLC diseases, to understand the regulated serum proteins in terms of their biological relevance, such as molecular activities, and to establish a database of serum indicators for NSCLC. Secondly, to validate our discovery effort, we selected two novel biomarker candidates -A1BG and LRG1-, by the means of traditional verification approaches, such as conventional western blotting and/or tissue microarray (TMA), as well as MRM measurements in larger sample cohorts.

## Methods

### Ethics and Collection of serum samples

Blood samples from newly diagnosed patients were obtained before any treatment in the Department of Thoracic Surgery at Shanghai Cancer Hospital. The inclusion criteria of subjects consisted of confirmed diagnosis of NSCLC, no distant metastasis or other surgical contraindications. The written informed consents were provided by all the individuals involved in this study. Anonymous samples from the patients were randomly selected. This study is approved by institutional review board of Fudan University Shanghai Cancer Center.

Serum samples were collected and processed using the same standardized protocol. Briefly, blood samples were left to coagulate at room temperature for 30 min, centrifuged at 3,000 g for 20 min (4°C), and the supernatants were collected, made to aliquots, and stored in −80°C until assayed. Time interval between processing and freezing was no more than 2 h for each sample. None of the samples was thawed more than twice before analysis.

### Delipidation and albumin depletion in serum samples

The delipidation and albumin depletion in the discovery phase were modified according to previously reported protocols [11], [12]. Briefly, for each sample, 50 µL crude serum was diluted with 250 µL buffer (100 mM NaCl, 10 mM HEPES, pH 7.4), and centrifuged at 10,000 g for 30 min through a 0.22 µm filter (Millipore) to remove lipids. Next, 260 µL delipidated serum was precipitated with 180 µL pre-chilled ethanol (Sigma), incubated for one hour at 4°C with gentle mixing, and centrifuged at 16,000 g for 45 min. The albumin-removed pellets were collected, lyophilized, and resuspended in 100 µL lysis buffer containing 8 M urea, 4% CHAPS, 40 mM Tris-base, 65 mM DTT and protease inhibitor cocktail (Roche, Ltd.).

### In-solution protein digestion

Protein mixtures were incubated with 10 mM dithiothreitol (DTT) at 37°C for 2.5 h, and carbamidomethylated with 20 mM iodoacetamide (IAA) for 45 min at room temperature in darkness. The alkylated protein solutions were precipitated by five volumes of pre-cold acetone/ethanol (1∶1, v/v) with 0.1% acetic acid at −20°C for 12 h [13]. The protein pellets were resuspended in 50 mM ammonium chloride buffer (pH 8.3), and incubated with trypsin (25∶1, Promega) for 20 h at 37°C. Digested peptides were lyophilized and stored at −80°C for mass spectrometry analysis.

### Online two-dimensional MS/MS analysis by Integrated multidimensional liquid chromatography

The extensive fractionation for serum peptides was performed on our Integrated multidimensional liquid chromatography (IMDL) system as previously described [7]. Basically, a bi-phase integrated column was utilized to achieve on-line two-dimensional HPLC separation. This integrated column included a strong cation column (SCX, 320-µm i.d., 50-mm length, Column Technology Inc., CA) and a reversed-phase (RP) chromatography column (150-µm i.d., 100-mm length, Column Technology Inc.). The peptide mixture was firstly injected by the Surveyor autosampler (ThermoFinnigan, San Jose, CA) and then fractionated by 11 pH steps (pH 2.5, 3.0, 3.5, 4.0, 4.5, 5.0, 5.5, 6.0, 7.0, 8.0, 8.5) using a series of pH buffers (configured by 5 mM citric acid adjusted by NH_4_OH). Different pH buffers were applied into the integrated column using a full loop injection of 100 µL at 3 µL/min before the on-line RP-HPLC gradient. The RP-HPLC solvents used were 0.1% formic acid (v/v) aqueous (A) and 0.1% formic acid (v/v) acetonitrile (B), with the gradient of 2 to 40% mobile phase B in 115 minutes at 2 µL/min after split. The mass spectrometer used was a linear ion trap LTQ (Thermo). A voltage of 3.0 kV was applied to the ESI needle, and the normalized collision energy was 35.0. The number of ions stored in the ion trap was regulated by the automatic gain control. The spectra were acquired in the data-dependent mode, with the option that 10 most intense ions of each MS spectrum for MS/MS analysis. The dynamic exclusion function was set as follows; repeat count 3, repeat duration 0.5 min, and exclusion duration 1.5 min.

### Protein identification

The BioWorks™3.2 software suite was used to generate the peak lists of all acquired MS/MS spectra (default parameters), which were then automatically searched against the Human International Protein Index protein sequence database (version 3.22, containing 57,867 proteins), using the SEQUEST version 2.7 (University of Washington, licensed to Thermo Finnigan) searching program. To estimate the rate of incorrect identifications (false positives), all the filtered spectra were subjected to database searching against a composite database containing human protein sequences in both the forward (correct) and reverse (incorrect) orientation [14]. In all database searches, trypsin was designated as the protease, and only one missed cleavage was allowed. Maximal mass tolerance was set as 3 Da for the precursor ion and 1.0 Da for fragment ions. Carbamidomethylation (+57.0125 Da) was searched as a fixed modification on cysteine, and oxidation (+15.99492 Da) was set as a variable modification on methionine. A homemade software Buildsummary was used to delete the redundant data as previously defined [15].

Proteins were identified with stringent criteria. Specifically, we employed the conservative naïve target-decoy strategy, which estimates protein identification false discovery rate (FDR) analogously to peptide-spectrum match (PSM) FDR, i.e. by approximating the expected number of false positive protein identification by the number of decoy protein identification [16]. Thresholds for Xcorr according to preliminary PSM FDR lower than 1% and the ultimate Protein FDR lower than 5% were applied. All accepted SEQUEST result must have a ΔCn score of at least 0.1 regardless of charge state [17].

### Label free quantification by spectral counting and statistics

A simple label free quantification approach using spectral counting was used to profile serum proteome between NSCLC and control cases [18]. Peptide-sequence matches assigned to every protein group were summed together and taken as basis of relative quantification [13], [17]. Cases in each cohort were treated as NSCLC-related biological replicates. We calculated the relative enrichment factor, R_e_, defined as R_e_ = (n_f_/n)/(N_f_/N) to prioritize overexpressed and under-expressed proteins [19]. In this equation, n_f_ is the number of peptide hits of a protein in one sample, n is the total number of peptide hits in this sample, N_f_ is the total hits number of this protein in all samples, and N is the total number of peptide hits of all the proteins in all samples. On the basis of R_e_, we executed ANOVA and student T test, using a permutation test of 200,000 times in Pomelo II [20]. The Receiver Operating Characteristic (ROC) analysis was performed by pROC package in R [21]. In ROC analysis of a biomarker panel, we used logistic regression model to assess the outcome of the panel, which incorporated the candidates we want to include in the panel (i.e. using block entry of variables) along with probabilities. This modeling was performed in SPSS (v13.0). The predicted probabilities of occurrence of the event (such as cancer or normal) were incorporated as predictors in ROC plot; and Area under ROC curve (AUC) was calculated to estimate the power of the panel [22]. Hierarchical clustering analysis (HCA) and Principal component analysis (PCA) was performed by R software (http://www.r-project.org/), using quantile normalized values of logarithmic transformed spectral counts, that is, by transformation of log_2_(spectral counts +1) for each serum protein in every sample.

### Tissue microarray analysis (TMA)

Immunohistochemical (IHC) staining was carried out using tissue microarrays purchased from Shanghai Outdo Biotech Co.. In brief, formalin-fixed, paraffin-embedded sections from 90 NSCLC patients, consisting of 44 lung adenocarcinoma (AD), 38 squamous cell carcinoma (SCC), and 8 cases of other subtypes, were deparaffinized and rehydrated. Endogenous peroxidase was quenched with 3% H_2_O_2_ (v/v). After antigen retrieval and blocking, sections were incubated with rabbit polyclonal anti-A1BG (1∶100, LifeSpan Biosiences), rabbit polyclonal anti-USP1 (1∶25, Abgent), mouse monoclonal anti-Mucin5B (1∶25, Millipore) and mouse monoclonal anti-LRG1 (Dilution 1∶200, Abonva) at 4°C overnight. After a rinse with PBST solution, the sections were incubated sequentially with biotinylated secondary antibody and ABC reagent (Vectastain), followed by treatment with DAB solution (Shanghai Sangon Biological Engineering Technology & Service Co.). Finally, sections were counterstained with Hematoxylin QS (Vectastain). Among the 90 cases, only one slice was lost during the processing, resulting in 89 NSCLC sections valid with their paired non-tumor controls.

IHC slides were revised by two pathologists (X. L., and H.C.), blinded to all clinicopathologic data. Results were averaged by their independent evaluations, and scored by estimating the percentage (P) of cells showing characteristic staining (from undetectable level or 0%, to homogeneous staining or 100%) and by estimating the intensity (I) of staining (0, no visual staining, 1, weak staining; 2, moderate staining; or 3, strong staining). The percentage was determined by the proportion of all the positive cells, without intuitive judgments of cell types. The percentage values were then collapsed into a P score from 0 to 9, corresponding to the ranges from 0–5%, 5–15%, 15–25%, 25–35% to 85–100% finally. As we proposed in a previous study [13], the relative protein expression in histological level were scored by multiplying P score by the intensity value I, i.e. by the so-called quick score (Q) (Q = P×I; minimum = 0 and maximum = 27).

### Western blotting

We performed western blot analysis in 12 randomly selected triplets of normal, AD and SCC sera. Protein samples were resolved on a 12% polyacrylamide gel and transferred onto PVDF membrane (Pall Inc.). Rabbit polyclonal anti-A1BG (LifeSpan Biosiences) was diluted as manufacturer's instructions and used to probe the blot. Immunodection was realized using ECL plus reagents (Amersham Biosciences) and were exposed to x-ray film (Kodak). After final WB images acquired, an image software named Multi Gauge (v3.0, FUJI FILM CO. LTD) was used to extract the protein expression data by IOD value.

### LC-MRM measurement

Four isotopic peptides assigned to A1BG and LRG1 (two per each protein) were synthesized using standard Fmoc chemistry incorporated with pure, heavy [^13^C_6_] Leucine (Sigma-Aldrich). Unlabeled [^12^C] forms of each peptide were also synthesized (GL Biochem, China). All synthetic peptides were purified to >95% purity with the amount reported from vendor.

Peptides digested from 0.1 µL crude serum (for 70 NSCLC sample and 30 age-matched controls, **see Table S1**) were resolubilized in 0.1% formic acid, spiked with internal standard peptides, and separated by reversed phase micro high-performance liquid chromatography on a 1200 HPLC system coupled with a 6410 QQQ mass spectrometer (Agilent Technologies). Separation was performed at mobile phase flow rate of 1.5 µL/min with Buffer A (0.1% formic acid) and Buffer B (90% acetonitrile in 0.1% formic acid), on one C18 trap column (300 µm×5 mm, Agilent Technologies) followed by an analytical C18 column (150 µm×100 mm, Column Technology Inc., CA). The binary gradient consisted of 3–17% B in 2.5 min, 17–23% B in 25 min, 23–40% B in 5 min, 40–100% B in 5 min and at 100% B for 2.5 min. Data was acquired with a capillary voltage of 4000 V, drying gas of 300°C at 3.0 L/min, and nebulizer gas of 18 psi. Fragmentor voltage and collision energy (CE) were optimized with infusion of each peptide. In MRM experiments, cycle times did not exceed 1.2 sec and a minimum of 20 data points were collected per peak.

### Absolute quantification of A1BG and LRG1 serum levels

MRM data analysis was performed using Masshunter Qualitative software (Agilent). [^12^C]/[^13^C] peak areas were recorded by manual inspection of strict co-eluting behavior, taking all the [^13^C] transitions as reference. For A1BG and LRG1, the verifications of their abundances in different cohorts were achieved by all the transition pairs, but only the best transitions which harbored best linearity response was used for absolute quantification. The assay linearity were characterized by diluting a tryptic digest of one serum sample which was incorporated with light peptides to generate a range of endogenous analyte concentrations spanning 3^7^ and 3^8^-fold concentration range (method modified from Michael et al., [23]) for LRG1 and A1BG reference peptides respectively. The amount of [^12^C] peptide was measured by the concentration point at which the area ratios of [^12^C]/[^13^C] were closest to 1 (1.029 for A1BG and 0.922 for LRG1). Area ratios were used to calculate endogenous concentrations of target proteins in sera, by formula fitted the linearity curve. Inter-assay coefficient of variation (CV) was evaluated by five separated processing and MRM replicates of a mixed sample, and limit of detection (LOD) was defined as the concentrations at which the S/N of the analyte is equal to 3 [24]. The limit of quantitation (LOQ) was empirically determined as the lowest analyte concentration which can keep the acceptable linearity (Pearson correlation, R>0.99) and can be measured with <20% CV [23].

## Results

### A high quality database of NSCLC associated serum proteins

**Figure 1:**
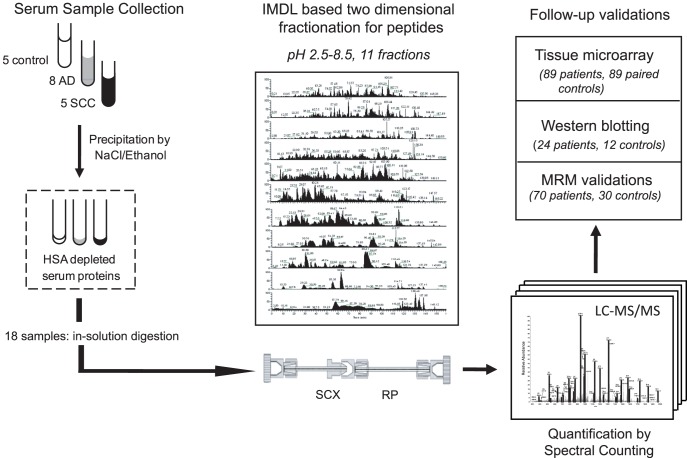
Experimental schema of the discovery and verification phases. Left panel, serum samples collection and depletion to remove the human serum albumin (HSA); Middle panel, IMDL fractionation which separates serum peptides by their pIs (2.5∼8.5) and hydrophobicity, with an example of HPLC-MS/MS chromatography showed for each pH step elution; Right panel, the number of NSCLC patients and the number of age-matched healthy controls in the verification phase by western blotting, TMA and MRM measurement. SCX, strong cation exchanger, RP reversed-phase chromatography. AD: Adenocarcinoma; SCC: Squamous cell carcinoma; NSCLC: Non-small cell lung cancer.

Pre-therapy sera from 13 NSCLC patients (8 ADs and 5 SCCs) and 5 healthy controls were processed for IMDL-MS/MS analysis (**Figure 1**). To unravel the complexity of serum proteome, we ameliorated a previous precipitation method to remove human serum albumin and utilized two-dimensional HPLC fractionation. The depletion process was fast and low-cost, and was quite reproducible and efficient, as demonstrated in **Figure S1**. A new series of buffers of eleven different pH values was used in IMDL, considering the complexity of human serum proteome (**Figure 1** **, middle panel**). Compared to a traditional one-dimensional RP-LC separation, proteome coverage was improved, considering that more than three times as many proteins were identified by IMDL under the same PSM FDR criteria (Data not shown).

With the respect to the growing concern over the confidence issue of blood proteins identified [25], we used the stringent criteria, naive target-decoy FDR on protein level to qualify serum proteome [16]. Collectively, 629,804 peptide hits of 4,456 unique peptides, assigning to 647 proteins were identified from serum (protein FDR<4.6%) and incorporated into label free quantification. We then observed the XCorr distribution of peptide identification in serum proteome. As **Figure S2** indicated, all of the spectra had a XCorr higher than 2.25, and were dominated by identification of much higher scores for both charge 2+ and charge 3+ ions. To further ascertain the performance of target-decoy strategy and its derived FDR, another widely used protein identification workflow, Trans-Proteomic Pipeline (TPP) [26], was also applied to all the raw spectra coming from one healthy serum sample. All the PSMs with PeptideProphet ≥0.75 were retained and assigned to proteins. Moreover, 89.7% proteins in our identification result had a ProteinProphet ≥0.9, and about 65.1% proteins had a ProteinProphet equaled 1. In contrast, if we retained the decoy tag in TPP, serum proteins with ProteinProphet ≥0.9 had a FDR higher than 10.7%. These suggested the fairly high confidence of our serum proteome.

### Profiling and biomarker screening in NSCLC serum proteome

We next estimated the dynamic range of the label free profiling. A significant correlation between protein spectral counts and their known corresponding concentrations [27], [28] was obtained (**Table S2**). Thus, our proteomic approach allowed opportunities of identification and relative quantification of serum proteins across six orders of magnitude (as low as several nanograms per milliliter).

**Figure 2:**
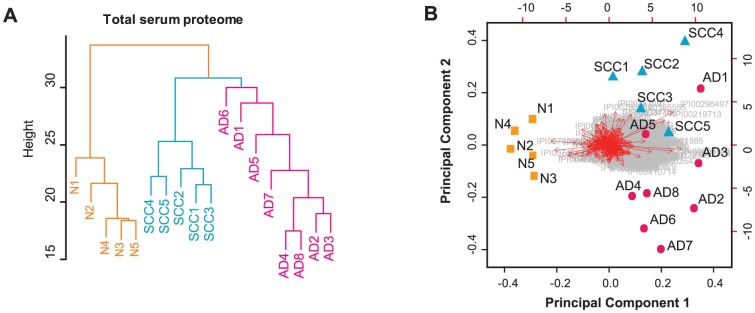
HCA and PCA of total serum proteomic data between samples. (A) Hierarchical clustering analysis and (B) Principal component analysis of all the 647 proteins quantified across the 18 serum samples. Ends of red arrows in (B) represent the PCA results for each protein.

To establish a NSCLC associated serum proteome, we started with performing Hierarchical clustering analysis (HCA) and Principal component analysis (PCA) on the total proteome to address that, if substantial serum proteins were regulated due to NSCLC occurrence. HCA and PCA were both performed on the spectral counting information of all 647 serum proteins (see method). The HCA implied that NSCLC and normal subjects were incorporated into two big clusters (**Figure 2A**). Similarly, in PCA analysis, NSCLC sera could be separated from 5 normal controls by only one principal component, and the variation in the cancer group was much more than that in the normal group (**Figure 2B**). Because the MS analysis of control cases were inserted randomly into the sequencing runs of 13 NSCLC sera, the pre-analytical factors should be uncertain and less significant than the cancer phenotype. Though the relative positions of every NSCLC case in HCA and PCA were not the same, we observed moderate divergence between AD and SCC patients. To answer if the separation between cohorts was coming from only one or two significantly changed abundant proteins, we also employed PCA for all the protein identities (see the red arrows in Figure 2B). This “bi-plot” suggested that sizeable proteins, rather than a small number of proteins, contributed to the separation.

**Figure 3:**
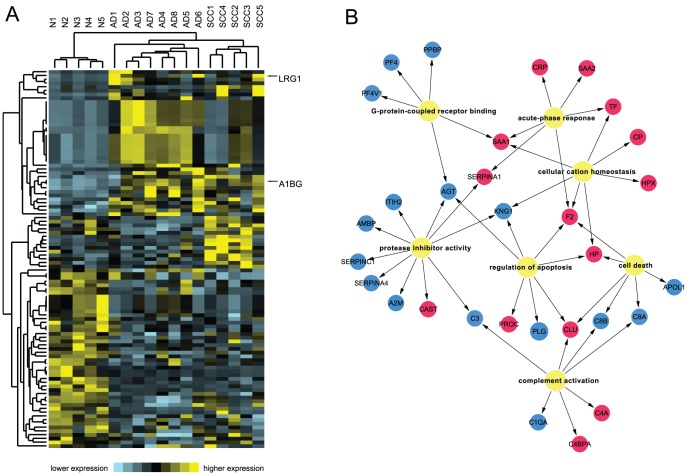
A high quality serum proteome associated with NSCLC. (A) Hierarchical clustering heatmap of 101 significantly altered proteins. Expression values are shown as a color scale with higher values represented by yellow and lower represented by blue. (B) Gene Ontology biological processes significantly enriched in 101 protein set. Gene symbols in blue or red circles (could be referred in Table S3) indicate the corresponding proteins in down- or up-regulated in NSCLC sera.

Since the identified serum protein might hold opportunities to delineate or predict NSCLC, permutated ANOVA and student T test between cohorts were employed. A total of 101 proteins were filtered as significantly differential proteins (p<0.01, see details in **Table S3**) and their relative enrichment pattern as a heatmap was shown in **Figure 3A**. Among 101 proteins filtered, more than 25% were previously indicated as NSCLC biomarker candidates. This significant consistency strongly validated our experimental approach in the discovery phase. Of note, most of the previous studies were based on 2D gel and reported much less differential proteins than our result [29], [30], [31]. Also, our 101 protein set successfully covered a substantial ratio of the candidates reported from a previous laborious work [32], which combined extensive multi-dimensional liquid chromatography and two-dimensional difference gel electrophoresis for each chromatography fraction. These evidences demonstrated that our online IMDL fractionation was not only convenient but also relatively sensitive in screening potential blood biomarkers. Besides the reported NSCLC candidate markers, we found sizeable noteworthy proteins closely related to other cancers, such as alpha-1B-glycoprotein [33], Complement C1q subcomponent subunit A [34], fibrinogen alpha/gamma chain [35], [36], fibulin-1 [37], platelet factor 4 and its variant [38]. Still, there were several proteins which harbored very good statistics, but lacked previous evidence to link them to cancer status, such as Protein AMBP (alternative name, alpha-1-microglobulin), Multivesicular body subunit 12B and V-type proton ATPase 116 kDa subunit. Interesting proteins that directly linked to a cancerous state (either NSCLC or other cancer in human) by literature mining were listed in **Table 1**.

**Table 1:** Serum proteins potentially relevant to NSCLC confirmed by IMDL and label free quantification in the discovery phase.

Protein Name	Gene symbol	IPI ID	SwissPort/Trembl	Normalized average peptide hits^a^			Referrences of NSCLC^b^
				N	AD	SCC	
**Up-regulated in AD and SCC**							
**Alpha-1-antitrypsin**	SERPINA1	IPI00553177	P01009	1396.7	2393.4	2460.0	[31], [56]
**C4b-binding protein alpha chain**	C4BPA	IPI00021727	P04003	164.8	264.5	264.1	[57], [58]
**C-reactive protein**	CRP	IPI00022389	P02741	0.0	15.2	3.5	[32]
**Haptoglobin**	HP	IPI00641737	P00738	337.2	764.3	693.3	[29], [32], [56], [57]
**Haptoglobin-related protein**	HPR	IPI00607707	P00739	93.4	369.5	295.9	[32]
**Hemoglobin subunit alpha**	HBA1/HBA2	IPI00410714	P69905	6.8	38.3	21.5	[30]
**Hemopexin**	HPX	IPI00022488	P02790	374.2	703.3	680.5	[29]
**Leucine-rich alpha-2-glycoprotein**	LRG1	IPI00022417	P02750	24.7	73.6	47.4	[32]
**Prothrombin**	F2	IPI00019568	P00734	156.4	185.8	203.9	[32]
**Serotransferrin**	TF	IPI00022463	P02787	2076.6	2781.6	2931.8	[56]
**Serum amyloid A protein**	SAA1	IPI00552578	P02735	2.2	87.8	51.3	[29], [59]
**Serum amyloid A2**	SAA2	IPI00006146	P02735	0.6	88.9	37.4	[60]
**Transthyretin**	TTR	IPI00022432	P02766	326.1	507.0	779.5	[30], [31], [60]
**Up-regulated in AD**							
**Complement component 4a**	C4A	IPI00744893	P0C0L4	55.4	99.5	53.8	[32]
**IGHA1 protein**	IGHA1	IPI00166866		288.3	516.6	366.4	[58]
**Ig alpha-2 chain C region**	IGHA2	IPI00641229	P01877	153.5	355.6	253.4	[58]
**IGHA2 protein**	IGHA2	IPI00644497		45.8	170.0	109.7	
**Up-regulated in SCC**							
**Apolipoprotein C-III**	APOC3	IPI00657670	P02656	1.0	3.5	5.3	[31]
**Ceruloplasmin**	CP	IPI00017601	P00450	167.1	61.7	179.2	[57]
**Clusterin**	CLU	IPI00291262	P10909	63.2	64.5	86.6	[32], [58]
**Up-regulated in N**							
**Alpha-2-macroglobulin**	A2M	IPI00478003	P01023	3356.6	2808.8	2802.3	[32]
**Angiotensinogen**	AGT	IPI00032220	P01019	235.8	127.6	121.4	[32]
**Antithrombin-III**	SERPINC1	IPI00032179	P01008	747.5	341.0	587.6	[29]
**Apolipoprotein L1**	APOL1	IPI00186903	O14791	28.9	11.7	21.5	[61]
**Complement C3**	C3	IPI00783987	P01024	3380.1	3341.6	2639.9	[32], [58]
**Gelsolin**	GSN	IPI00026314	P06396	101.3	47.5	67.2	[32], [61]
**Plasminogen**	PLG	IPI00019580	P00747	370.3	247.6	279.3	[32]
**Tetranectin**	CLEC3B	IPI00009028	P05452	8.7	1.9	3.7	[32]

a Averaged PSMs (peptide hits) in Normal (N), AD and SCC group.

b Reference listed were closely related to NSCLC and all reported the identical up or down regulation trend with our results.

### Biological Processes enriched in differential serum proteome of NSCLC

We next sought to characterize the 101 proteins in context of their biological functions. By comparing GO biological process annotated by BINGO, we found several processes were significantly enriched (adjust p<0.05 after BH correction, **Figure 3B**). Specifically, processes of acute-phase responses (p = 5.26E−10) and cellular cation homeostasis (p = 1.31E−6) in circulatory system tended to be up-regulated in cancerous sera. Processes related to complement activation (p = 1.28E−7), cell death (p = 1.15E−2) and apoptosis (p = 1.27E−3) were also significantly regulated. Besides, we found a number of protein activities not previously known to reside in lung cancer, such as protease inhibitor and G-protein-coupled receptor binding activity, whose relevant proteins were mostly down-regulated (p = 1.56E−11 and 3.24E−6). Collectively, these observations implicate NSCLC not only in the acute phase responses and immunity, but also in specific signal transductions and death processes.

### TMA and scoring to validate the histological expression A1BG, USP1 and mucin 5B in NSCLC tissues

**Figure 4:**
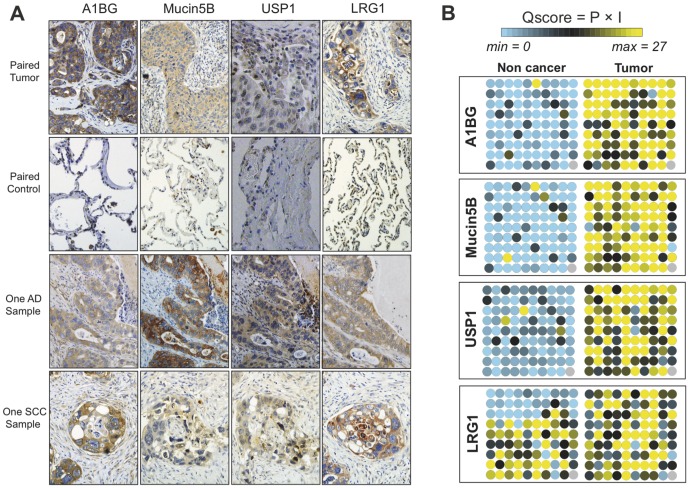
TMA assisted validation for histological biomarkers. (A) Representative IHC cases with higher expression level in NSCLC sections. One SCC and one AD sample are shown to suggest different Q scores can be obtained by the four proteins; magnification 200×. (B) Virtual result of TMA. Q scores are calculated considering both percentage of positive cells and stain intensity and shown as a color scale with higher Q represented by yellow (maximum = 27) and lower Q represented by blue (minimum = 0). The grey spot represents the only one case lost during the processing.

To show that some, if not all, of the proteins of interest are indeed associated with NSCLC risk, in the verification phase we utilized different validation approaches. Because high concordance was previously observed between TMA measurement and RT-PCR [39] or proteomic profiling [40], we asked whether the TMA could enable a fast, rigorous and semi-quantitative estimate of the histological biomarkers. In this section, besides alpha-1B-glycoprotein (A1BG) and Leucine-rich alpha-2-glycoprotein (LRG1) which are of mid-range abundance in the serum, we chose a low abundant serum protein ubiquitin carboxyl-terminal hydrolase 1 (USP1) that was detected in NSCLC sera. In addition, because cell line lysate could be biologically more close to tissue sample, we selected on candidate mucin-5B that detected in the NSCLC cell level according to a dataset in our previous study [17]. Our TMA towards these four proteins provided a homogeneous and synchronous profiling of on 90 paired NSCLC sections. All tested proteins demonstrated higher expression level in NSCLC tumor sections (**Figure 4A**). To closely access the differential level between NSCLC and their adjacent non-tumor sections, the IHC staining intensity (I, 0–3) and the percentage (P, 0–9) of positive stained cells were distributed (**Figure 4B** and **Figure S3**). Most tumor sections were characterized with higher intensity (I≥2) and more positive cells (P>60%). One of the interesting outcomes was the direct observation of varied levels of different proteins on the same patient specimen (**Figure 4A**), yielding the hypothesis that stronger classifying power could be achieved by combining histological candidates. The result of this section shows the heightened levels of A1BG, LRG1 and USP1 in sera might be partially ascribed to their overexpression in the tumor tissues and shows that all of the four proteins have the potential to be histological biomarkers for NSCLC diseases.

### Western blotting verification of A1BG as NSCLC associated serum candidate

**Figure 5:**
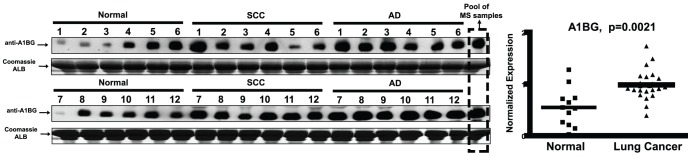
Workflow of western blotting assisted verification for A1BG. Western blot images across 12 ADs, 12 SCCs and 12 control sera. Coomassie stained Albumin (ALB) was shown as the loading controls. IOD values were extracted and normalized by this calibration sample, suggesting the differential distribution of A1BG serum levels between normal and lung cancer cases.

Because of the antibody issues or the limited MRM sensitivity, we could not detect USP1 and muscin 5B in NSCLC serum samples, we then focus on the validation of A1BG and LRG1 in the following experiments. Western blotting (WB) validation was done only for A1BG (**Figure 5**) due to antibody compatibility. To find effective way to do WB for proteomic verification, i.e. to profile the protein expressions across a large sample scale after discovery phase by MS analysis, we recruited one “calibration sample” which pooled all the 18 MS samples in the discovery phase with equal constitutes. This pooled sample was identically treated with other samples in every run of WB, providing an internal standard to link different WB membranes. After final WB images acquired, an image software was used to extract the protein expression data by IOD value. The expression levels in each WB run thus could be normalized by this calibration sample and be compared, suggesting that A1BG indeed had a significant differential expression between NSCLC and control group (p = 0.0021).

### MRM assay for the absolute quantification of AIBG and LRG1 in NSCLC blood samples

Taking the merits of the current targeted proteomics for clinical use, we next attempted to configure a MRM assay coupled with stable isotope dilution mass spectrometry (SID-MS) to measure the absolute concentrations of A1BG and LRG1. Two unique peptides of A1BG and LRG1 were selected, considering their relative detectable ability observed in our shotgun proteomic data. The non-specific isotopic incorporation was minimal (<0.2%, data not shown) and was not considered.

**Figure 6:**
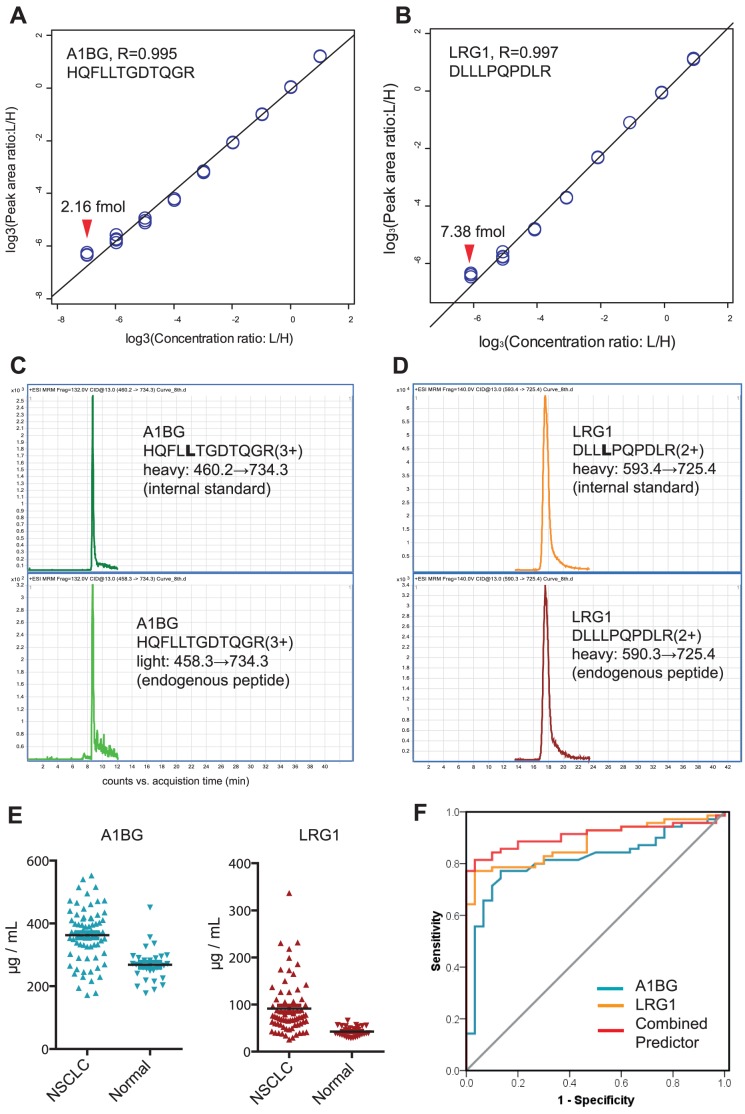
The MRM measurement of serum concentrations of A1BG, LRG1 and their association with NSCLC. (A–B) MRM Intensities of selected reference peptides of A1BG and LRG1 both showed good linear correlation with on-column abundance. The x-axis represents base-3 logarithm of ratios of spiked light and heavy isotopic peptides, with the y-axis corresponding to the observed peak area ratios in base-3 logarithmic scale. Red triangles suggest the limit of linear quantification (LOQ) of each peptide. Note that these two peptides were used to report the absolute concentration, with comparison to another two less-optimal peptides shown in Figure S4. (C–D) The chromatography peaks of the best transitions of two peptides for A1BG and LRG1. Note that the Signal-to-Noise ratios are sufficient for quantification. (E) The distribution of serum levels of A1BG and LRG1 between cancer and control groups. (F) ROC analysis suggested over-expressed A1BG and LRG1 were associated with NSCLC and their combined panel could provide the better discriminative performance than each of the protein.

Eight pairs of light-to-heavy MRM transitions for A1BG and seven pairs for LRG1 were optimized with MS parameters to achieve the highest signal transmission (**Table S4**). Both A1BG peptides harbored the great linear correlations with MRM intensities, as implicated in the 3-fold dilution curves (R = 0.993 and 0.995 for A1BG; R = 0.997 and 0.986 for LRG1 respectively, **Figure 6A–B** **and Figure S4**). According to the results of absolute quantification by the heavy labeled peptides, good agreement of relative quantification was obtained by using different MRM transitions of the two peptides per each protein. However, we found HQFLLTGDTQGR of A1BG and DLLLPQPDLR of LRG1 offered better limits of linear quantification than the other two less-optimal peptides (**Figure S4**), touching the limit of quantifying 2.16 fmol and 7.38 fmol peptides on column (**Figure 6A**). This implicated that concentrations down to µg/mL for A1BG and LRG1 in serum could be confidently quantified, with the very decent signals observed from their endogenous peptides (**Figure 6C–D**). Another expected benefit of our MRM assay is the high reproducibility with extremely low inter-assay CV of 2.23% for A1BG and 1.90% for LRG1 quantification (**Table S5**), due to the less experimental variations introduced by non-fractionation method in our protein sample preparation. These suggested that our MRM assay was sufficient to measure the absolute serum concentrations of A1BG and LRG1 in 100 cases reproducibly, which were all listed in **Table S6**. The MRM measurement showed consistency with western blotting result for A1BG and confirmed the clinical significance for both proteins (**Figure 6E**, p<0.0001, student's t-test). The Area Under the Curve (AUC) in Receiver operating characteristic curve (ROC) analysis was 0.816 (95% CI, 0.729 to 0.903) for A1BG and 0.880 (0.815 to 0.945) for LRG1, verifying the association between overexpressed two proteins and NSCLC diseases (**Figure 6F**). Intriguingly, the biomarker panel combined A1BG and LRG1 created by logistic regression had an AUC of 0.909 (0.851 to 0.968), higher than that of each protein, indicating the combination power of the two proteins to discriminate healthy and cancer sera (**Figure 6F**). Finally, we surveyed if the over-expressions of A1BG and LRG1 were correlated with any patient demographics. We found that the blood levels of the two proteins were independent of the histological subtypes (AD vs. SCC; A1BG, *P* = 0.380; LRG1, *P* = 0.512), disease stages (stage I–II vs. III–IV; A1BG, *P* = 0.846; LRG1, *P* = 0.314), smoking histories (Yes vs. No; A1BG, *P* = 0.183; LRG1, *P* = 0.456), sex (A1BG, *P* = 0.178; LRG1, *P* = 0.999) and age (Pearson correlation coefficient; A1BG, R = −0.198; LRG1, R = 0.268), but only showed the strong relevance with the risk of lung cancer.

## Discussion

In this study, to deal with the analytical difficulties of blood proteomics, we utilized the current clinical shotgun and targeted proteomics to discover and verify NSCLC blood biomarkers. Using a label free quantification method in the discovery phase, we reported a deeper proteomic profiling towards more than 600 proteins by the extensive two dimensional fractionations of the serum peptides. The fast, cost-efficient depletion and the deep, reproducible IMDL fractionations provided state-of-art proteomic discovery experiments, which can analyze 10–15 samples with in a still reasonable time (2–3 weeks). A high quality NSCLC associated serum protein database consisting of 101 proteins was created. This dataset contains substantial proteins that supported by previous similar works. The GO biological process annotation of this dataset seems not only revealed some acute phase reactions but also interesting cancer related pathways (**Figure 3B**).

The primary objective of verification is to screen potential biomarkers to ensure that only the highest quality candidates from our proteomic discovery phase are taken forward into pre-clinical validation. To quickly expedite the classification power, we elaborately configured different workflows. Inheriting semi-quantitative nature of WB, our WB assisted workflow allowed a global profiling of candidates on a theoretically unlimited number of samples. The WB workflow could be efficient in the very first phase of biomarker screening after MS analysis. One limitation of this workflow might be the sensitivity of WB itself for blood analysis, because the amounts of targeted serum proteins can be very low in the limited loading volume. In histological level, the use of TMAs is relatively new for lung cancer research. The superiority of TMA is its ability to efficiently analyze large numbers of NSCLC specimens in a methodologically uniform way. One arguable issue is that whether the paired control specimens could represent the real normal tissues, which remains unproved in current IHC studies. Because antibody reagents for a clinical-grade immunoassay only exist for a small number of candidates, alternative approaches are required to credential novel candidates quickly and effectively. We configured a MRM assay coupled with stable isotope dilution mass spectrometry (SID-MS) to measure the absolute concentration of A1BG and LRG1 in the sera. The blood assays of the two proteins (**Table S4**) are transferrable for future studies.

This study aims to discover diagnostic biomarkers for NSCLC. However, to define the biological relationship between the biomarkers we supported and NSCLC is beyond the scope of this study. In histological level, besides our previous study [17], the highly glycosylated protein mucin-5B was previously reported to up-regulated in endometrial adenocarcinomas [41], and was already included in the lists of significantly changed proteins in two proteomic analysis of lung squamous carcinoma tissues [42], [43]. Our IHC data presents credential to its differential expression in both SCCs and ADs. USP1 had never been suggested to function in NSCLC, but this protein was believed to serve as a negative regulator of DNA damage repair [44]. USP1 also involved in translesion synthesis (TLS) by deubiquitinating proliferating cell nuclear antigen (PCNA), which was indicated to correlate with proliferative activity in NSCLC [45]. The molecular association of Mucin-5B and USP1 with NSCLC tumor status remains to be illustrated.

Previously, the associations between A1BG expression in bio-fluids (pancreatic juice, urine and serum) and the presence of pancreatic cancer, bladder cancer and breast cancer has been evidenced. Tian et al. used difference gel electrophoresis (DIGE) and tandem mass spectrometry to compare the pancreatic juice between 9 pancreatic ductal adenocarcinoma (PDAC) patients and 9 cancer-free controls. They found the up-regulation of A1BG in pancreatic juice and PDAC tissues, which was further validated by western blot and Immunochemistry [46]. Using a N-linked glycoprotein enrichment technology by lectin affinity chromatography and a sample set of 5 patients, Kreunin et al. discovered A1BG as a human urine protein that only detected in bladder cancer bearing patient samples but in none of the samples obtained from non-tumor-bearing individuals [47]. In a study of breast cancer, Zeng et.al applied multiple lectin affinity chromatography coupled with 1D SDS-PAGE, isoelectric focusing as a comprehensive serum proteomics platform and analyzed 5 patient samples and 5 controls. It is indicated that A1BG, together with Complement C3, alpha-1-antitrypsisn and transferrin as potential biomarkers for further study [48]. In a recent report, Li et al. utilized a multiplexed bead-based antibody-lectin assay to simultaneously profile the glycosylation patterns of serum proteins for the biomarker discovery. According to their result, a decline of A1BG signal was found to distinguish 20 patients with stage III and IV pancreatic cancer from normal controls and renal cell carcinoma samples [49]. To the best of our knowledge, our study here is the first report that links the A1BG blood/tissue expression with lung cancer diseases, and we validated this linkage in a cohort of about 100 patients, which is much larger than the sample sets in above previous studies.

LRG1 is a secreted glycoprotein of unknown function. Based on mRNA analysis, it was predicted to be produced by liver cells [50] and by neutrophils [51]. In previous literatures, LRG1 showed promise to indicate inflammation in a mouse model, granulocytic differentiation [51], and was listed as one of the altered proteins in cancer proteomic studies, such as hepatocellular carcinoma [52], pancreatic cancer [53], and lung cancer [32]. We herein further validated the association of LRG1 up-regulation and NSCLC diseases in both tissue and blood levels using much larger sample cohort than previous studies [32]. Although LRG1 has been shown to bind cytochrome c and has been hypothesized to play a role in cell survival [54], we deemed important to search other explanations for its overexpression in NSCLC cases due to its abundance in serum.

Moreover, we did not observe significant correlations between either A1BG or LRG1 serum expressions and the patient demographics such as subtype, tumor stage or smoking history. This could suggest that more samples might be needed to uncover the correlation, or could suggest that A1BG and LRG1 could be released into the circulation at the tumor onset stage (very early stage) and thus is generally associated with systematic cancer status.

It is interestingly to notice that, though we harnessed the current, advanced clinical proteomic strategies, most proteins we identify are still of moderate abundance in the blood, such as A1BG and LRG1. Therefore, the overexpression of both proteins could only be partially ascribed to the tumor releasing process even with the positive IHC results. This underscores the difficulty of direct biomarker search in the blood sample. More elaborated fractionation experiments such as sub-proteome enrichment (e.g. N-linked glycoproteome [55]) are needed in the future. Alternative discovery sources such as cancer secretomes, blood cells and other accessible biofluids should also be necessary and useful for a long time. Finally, the specificity of the NSCLC biomarker reported here still need to be explored in the future studies, with the comparison of other cancers. The further studies of validating the discovered diagnostic biomarker candidates with other clinical purposes, such as prognosis refinement and personalized medicine or therapeutics are currently underway in our group.

## Supporting Information

Table S1 **Patient demographics of 100 serum samples for A1BG measurement.**
(DOC)

Table S2 **Thirty-six proteins with spectral counting data (number of PSMs/protein) in the discovery phase and known concentrations of proteins in human plasma.**
(DOC)

Table S3 **A total of 101 proteins filtered as significantly differential proteins.** Note that all the reference listed were closely related to NSCLC and reported the identical up or down regulation trend with our results. ^a^ Averaged PSMs (peptide hits) in Normal (N), AD and SCC group. ^b^ Raw PSMs identified in A549 and H1703 cells and conditioned media.
(DOC)

Table S4 **The final MRM transitions of A1BG and LRG1 with their optimized fragmentor and collision energy.** ^a^ **L** and **A** represent [^13^C_6_] Leucine and [^13^C_3_] Alanine for heavy isotopic peptides respectively. ^b^ * means the best transition pairs for the absolute quantification of the protein. ^c^ Both Q1 and Q2 were set at Unit Resolution.
(DOC)

Table S5 **The performance of the final MRM assay.** Note that extremely low inter-assay CVs can be achieved due to the less experimental variations in analyzing non-depletion and non-fractionation sera. ^a^ **L** represents [^13^C6] Leucine and [^13^C3] Alanine for heavy isotopic peptides respectively. ^b^Determined as the lowest concentration touched with S/N≥3. ^c^Defined as lowest concentration in the linearity curve (R>0.99) with CV<20%. ^d^Inter-assay CV was determined by a pooled cancer serum sample in 5 experimental replicates.
(DOC)

Table S6 **The serum levels of A1BG and LRG1 in all the 100 samples measured by MRM assays.** Normal, age-matched normal controls; AD: Adenocarcinoma; SCC: Squamous cell carcinoma.
(DOC)

Figure S1 **The albumin depletion for serum proteome.** (A) Eighteen serum samples of the same starting volume were loaded for one dimensional electrophoresis. The resultant albuminome supernatant and HSA depleted pellet fractions decently showed quite analogous constitutes between individuals. (B) The streamlined protocol was also applied to one healthy serum and repeated for four times separately. Identical patterns were also observed from both supernatant and pellet fractions between these technical replicates.
(TIF)

Figure S2 **Xcorr distribution and peptide identification in serum proteome.** (A) all of the spectra had a Xcorr higher than 2.25, and were dominated by identification of much higher scores of charge 2+ and charge 3+ ions. (B) Comparison between naive target-decoy protein FDR and Trans-Proteomic Pipeline (TPP) [26]. TPP was applied to all the raw spectra coming from one healthy serum. All the PSMs with PeptideProphet ≥0.75 were retained and assigned to proteins. Notably, 89.7% proteins in our identification result (by protein FDR) have a ProteinProphet ≥0.9. The proteins with a ProteinProphet of zero were all identified by multiple PSMs (33-25953 matches), and may be caused by the different peptide-protein group assignment priorities between TPP and Buildsummary. In contrast, if we retained the decoy tag in TPP, the final serum proteins with ProteinProphet ≥0.9 has a protein FDR equaled 10.7%, suggesting the fairly high confidences of our serum proteome.
(TIF)

Figure S3 **The distribution of the IHC staining intensity (I) and percentage (P) of positive stained cells.** Both of them characterized most tumor sections with higher intensity (≥2) and more positive cells (>60%).
(TIF)

Figure S4 **The MRM assays of the two less optimal reference peptides (compared to** **Figure 6** **) for A1BG and LRG1 measurements.** MRM Intensities of two peptides both showed good or modest linear correlation with on-column abundance. The x-axis represents base-3 logarithm of ratios of spiked light and heavy isotopic peptides, with the y-axis corresponding to the observed peak area ratios in base-3 logarithmic scale. Red triangles suggest the limit of linear quantification (LOQ) of each peptide. (C–D) The chromatography peaks of the best transitions for two less-optimal peptides. L in bold indicates the pure, heavy [^13^C_6_] Leucine.
(TIF)
