# Supplementary material for: Integrative Proteomics and Tissue Microarray Profiling Indicate the Association between Overexpressed Serum Proteins and Non-Small Cell Lung Cancer
Source: PLoS One. 2012 Dec 19;7(12):e51748. doi: 10.1371/journal.pone.0051748 (PMC3526638; doi:10.1371/journal.pone.0051748)
Supplement: Table S1 — Patient demographics of 100 serum samples for A1BG measurement. (DOC) [file pone.0051748.s001.doc]

**Table S1. Patient demographics of 100 serum samples for A1BG measurement.**

|  | **NSCLC**  **(n=70)** | **Control (n=30)** |
| --- | --- | --- |
| ***Age***, years | 62.6±8.81 | 55.7±10.5 |
| Range | 41-74 | 37-76 |
| ***Gender*** |  |  |
| Male | 50 | 15 |
| Female | 20 | 15 |
| ***Stage*** |  |  |
| I | 9 |  |
| II | 12 |  |
| III | 48 |  |
| IV | 1 |  |
| ***Histology*** |  |  |
| AD | 15 |  |
| SCC | 45 |  |
| ***Smoking*** |  |  |
| Yes | 47 | 12 |
| No | 23 | 18 |
