# Supplementary material for: Integrative Proteomics and Tissue Microarray Profiling Indicate the Association between Overexpressed Serum Proteins and Non-Small Cell Lung Cancer
Source: PLoS One. 2012 Dec 19;7(12):e51748. doi: 10.1371/journal.pone.0051748 (PMC3526638; doi:10.1371/journal.pone.0051748)
Supplement: Table S2 — Thirty-six proteins with spectral counting data (number of PSMs/protein) in the discovery phase and known concentrations of proteins in human plasma. (DOC) [file pone.0051748.s002.doc]

**Table S2. Thirty-six proteins with spectral counting data (number of PSMs/protein) in the discovery phase and known concentrations of proteins in human plasma.**

| **Protein Name** | **Peptide hits** | **Concentration(pg/ml)^a^** |
| --- | --- | --- |
| Hepatocyte growth factor receptor | 1 | 1.00E+05 |
| Metalloproteinase inhibitor 2 | 1 | 3.40E+04 |
| Plasminogen activator inhibitor 1 | 1 | 4.20E+04 |
| Transforming growth factor beta-1 | 1 | 1.40E+04 |
| Matrix metalloproteinase-2 | 2 | 1.45E+05 |
| Fructose-bisphosphate aldolase A | 3 | 1.70E+05 |
| Fructose-bisphosphate aldolase C | 4 | 2.00E+04 |
| lactotransferrin | 14 | 2.70E+05 |
| insulin-like growth factor binding protein 3 | 36 | 2.50E+06 |
| Adiponectin | 37 | 3.93E+06 |
| Cholesteryl ester transfer protein | 47 | 1.90E+06 |
| Vitamin K-dependent protein C | 52 | 3.70E+06 |
| Coagulation factor IX | 78 | 5.10E+06 |
| Apolipoprotein E | 83 | 4.00E+07 |
| Coagulation factor V | 105 | 6.60E+06 |
| C-reactive protein | 144 | 8.22E+06 |
| Serum amyloid P-component | 230 | 3.42E+07 |
| Coagulation factor XII | 377 | 3.00E+07 |
| Fibrinogen alpha chain | 603 | 1.00E+07 |
| Apolipoprotein A-I | 704 | 3.80E+05 |
| Serum amyloid A protein | 908 | 3.86E+07 |
| Leucine-rich alpha-2-glycoprotein | 941 | 2.20E+07 |
| Clusterin | 1225 | 3.70E+08 |
| Vitronectin | 1238 | 2.60E+08 |
| Ceruloplasmin | 2037 | 2.80E+08 |
| Angiotensinogen | 2554 | 1.50E+06 |
| C4b-binding protein alpha | 4247 | 3.80E+08 |
| Alpha-1B-glycoprotein | 4856 | 2.70E+08 |
| Plasminogen | 5154 | 1.00E+08 |
| Transthyretin | 9251 | 1.18E+08 |
| Haptoglobin | 11385 | 6.20E+08 |
| Complement C4-A | 14145 | 2.70E+08 |
| Alpha-1-antitrypsin | 37652 | 1.90E+09 |
| Serotransferrin | 44416 | 4.00E+09 |
| Alpha-2-macroglobulin | 51434 | 1.80E+09 |
| Complement C3 | 63158 | 1.30E+09 |

^a^  Concentrations were known according to two references[1,2]，a website ( <http://www.rulesbasedmedicine.com/case-studies/figures/serum_or_plasma-table1.html>) and concentration data in this work.

**Reference**

1. Polanski M, Anderson NL (2007) A list of candidate cancer biomarkers for targeted proteomics. Biomark Insights 1: 1-48.

2. Anderson L (2005) Candidate-based proteomics in the search for biomarkers of cardiovascular disease. J Physiol 563: 23-60.
