# Supplementary material for: Integrative Proteomics and Tissue Microarray Profiling Indicate the Association between Overexpressed Serum Proteins and Non-Small Cell Lung Cancer
Source: PLoS One. 2012 Dec 19;7(12):e51748. doi: 10.1371/journal.pone.0051748 (PMC3526638; doi:10.1371/journal.pone.0051748)
Supplement: Table S3 — A total of 101 proteins filtered as significantly differential proteins. Note that all the reference listed were closely related to NSCLC and reported the identical up or down regulation trend with our results. a Averaged PSMs (peptide hits) in Normal (N), AD and SCC group. b Raw PSMs identified in A549 and H1703 cells and conditioned media. (DOC) [file pone.0051748.s003.doc]

**Table S3. A total of 101 proteins filtered as significantly differential proteins.** Note that all the reference listed were closely related to NSCLC and reported the identical up or down regulation trend with our results.

| **Protein Name** | **Gene symbol** | **IPI ID** | **SwissPort/**  **Trembl** | **Normalized average**  **peptide hits^a^** | | | | **Referrences of NSCLC** |  |
| --- | --- | --- | --- | --- | --- | --- | --- | --- | --- |
|  |  |  |  | **N** | **AD** | **SCC** | |  |  |
| **Up-regulated in AD and SCC** | | | | | | | | |  |
| **Alpha-1-antitrypsin** | SERPINA1 | IPI00553177 | P01009 | 1396.7 | 2393.4 | 2460.0 | | Ref[1,2] |  |
| **Alpha-1B-glycoprotein** | A1BG | IPI00022895 | P04217 | 129.9 | 293.5 | 347.6 | |  |  |
| **C4b-binding protein alpha chain** | C4BPA | IPI00021727 | P04003 | 164.8 | 264.5 | 264.1 | | Ref [3,4] |  |
| **C-reactive protein** | CRP | IPI00022389 | P02741 | 0.0 | 15.2 | 3.5 | | Ref [5] |  |
| **Fibrinogen alpha chain** | FGA | IPI00021885 | P02671 | 7.4 | 39.5 | 51.9 | |  |  |
| **Fibrinogen gamma chain** | FGG | IPI00219713 | P02679 | 0.2 | 18.9 | 64.0 | |  |  |
| **Haptoglobin** | HP | IPI00641737 | P00738 | 337.2 | 764.3 | 693.3 | | Ref [1,3,5,6] |  |
| **Haptoglobin-related protein** | HPR | IPI00607707 | P00739 | 93.4 | 369.5 | 295.9 | | Ref [5] |  |
| **Hemoglobin subunit alpha** | HBA1/HBA2 | IPI00410714 | P69905 | 6.8 | 38.3 | 21.5 | | Ref [7] |  |
| **Hemoglobin subunit beta** | HBB | IPI00654755 | P68871 | 8.4 | 24.1 | 15.5 | |  |  |
| **Hemopexin** | HPX | IPI00022488 | P02790 | 374.2 | 703.3 | 680.5 | | Ref [6] |  |
| **Leucine-rich alpha-2-glycoprotein** | LRG1 | IPI00022417 | P02750 | 24.7 | 73.6 | 47.4 | | Ref [5] |  |
| **Multivesicular body subunit 12B** | FAM125B | IPI00552643 | Q9H7P6 | 0.7 | 12.6 | 8.2 | |  |  |
| **Myosin-reactive immunoglobulin light chain variable region** |  | IPI00384398 | Q9UL81 | 1.8 | 11.9 | 4.9 | |  |  |
| **Prothrombin** | F2 | IPI00019568 | P00734 | 156.4 | 185.8 | 203.9 | | Ref [5] |  |
| **Serotransferrin** | TF | IPI00022463 | P02787 | 2076.6 | 2781.6 | 2931.8 | | Ref [1] |  |
| **Serum amyloid A protein** | SAA1 | IPI00552578 | P02735 | 2.2 | 87.8 | 51.3 | | Ref [6,8] |  |
| **Serum amyloid A2** | SAA2 | IPI00006146 | P02735 | 0.6 | 88.9 | 37.4 | | Ref [9] |  |
| **Transthyretin** | TTR | IPI00022432 | P02766 | 326.1 | 507.0 | 779.5 | | Ref [2,7,9] |  |
| **41 kDa protein** |  | IPI00644018 |  | 83.5 | 214.1 | 241.1 | |  |  |
| **Up-regulated in AD** | | | | | | | | |  |
| **Complement component 4a** | C4A | IPI00744893 |  | 55.4 | 99.5 | 53.8 | | Ref [5] |  |
| **highly similar to Protein Tro alpha1 H,myeloma** |  | IPI00647704 | Q6ZW64 | 286.2 | 499.9 | 365.4 | |  |  |
| **highly similar to SNC73 protein** |  | IPI00386879 | Q96K68 | 282.2 | 517.3 | 373.8 | |  |  |
| **IGHA1 protein** | IGHA1 | IPI00166866 |  | 288.3 | 516.6 | 366.4 | | Ref [4] |  |
|  |  | IPI00430842 |  |  |  |  |  |  |  |
|  |  | IPI00449920 |  |  |  |  |  |  |  |
| **Ig alpha-2 chain C region** | IGHA2 | IPI00641229 | P01877 | 153.5 | 355.6 | 253.4 | | Ref [4] |  |
| **IGHA2 protein** | IGHA2 | IPI00644497 |  | 45.8 | 170.0 | 109.7 | |  |  |
|  |  | IPI00423461 |  |  |  |  |  |  |  |
| **Immunoglobulin alpha-2 heavy chain** | IGHA2 | IPI00784830 |  | 135.3 | 317.9 | 222.7 | |  |  |
|  |  | IPI00785067 |  |  |  |  |  |  |  |
|  |  | IPI00784950 |  |  |  |  |  |  |  |
|  |  | IPI00784969 |  |  |  |  |  |  |  |
|  |  | IPI00642017 |  |  |  |  |  |  |  |
|  |  | IPI00784758 |  |  |  |  |  |  |  |
| **Putative uncharacterized protein DKFZp686J11235** |  | IPI00426060 | Q6MZW0 | 274.9 | 498.2 | 349.0 | |  |  |
| **Putative uncharacterized protein DKFZp686G21220** |  | IPI00423460 | Q6N090 | 223.9 | 394.3 | 294.3 | |  |  |
| **Hypothetical protein** |  | IPI00423462 | Q6N092 | 278.9 | 510.7 | 385.2 | |  |  |
| **Up-regulated in SCC** | | | | | | | | |  |
| **Apolipoprotein C-III** | APOC3 | IPI00657670 | P02656 | 1.0 | 3.5 | 5.3 | | Ref [2] |  |
| **Calpastatin** | CAST | IPI00220859 | P20810 | 0.0 | 0.2 | 2.1 | |  |  |
| **Ceruloplasmin** | CP | IPI00017601 | P00450 | 167.1 | 61.7 | 179.2 | | Ref [3] |  |
| **Clusterin** | CLU | IPI00291262 | P10909 | 63.2 | 64.5 | 86.6 | | Ref [4,5] |  |
| **Fibulin-1** | FBLN1 | IPI00296534 | P23142 | 9.0 | 6.7 | 18.2 | |  |  |
| **Ig heavy chain V-III region HIL** |  | IPI00382488 | P01771 | 1.9 | 2.2 | 5.0 | |  |  |
| **Ig kappa chain V-I region Kue** |  | IPI00387096 | P01604 | 1.9 | 1.0 | 3.4 | |  |  |
| **Ig kappa chain V-I region Mev** |  | IPI00387105 | P01612 | 0.6 | 1.9 | 5.8 | |  |  |
| **Ig lambda chain V-I region NEWM** |  | IPI00382422 | P01703 | 2.3 | 3.3 | 5.1 | |  |  |
| **Tenascin-X** | TNXB | IPI00025276 | P22105 | 0.8 | 0.1 | 1.8 | |  |  |
| **V1-5 protein** | IGLV2-18 | IPI00553215 | Q5NV65 | 0.4 | 0.0 | 1.2 | |  |  |
| **Vitamin K-dependent protein C** | PROC | IPI00021817 | P04070 | 3.7 | 1.1 | 5.1 | |  |  |
| **12 kDa protein** |  | IPI00747345 |  | 0.2 | 0.1 | 1.3 | |  |  |
| **Up-regulated in N and SCC** | | | | | | | | | |
| **EGF-containing fibulin-like extracellular matrix protein 1** | EFEMP1 | IPI00029658 | Q12805 | 1.9 | 0.0 | 1.8 | |  | |
| **V2-19 protein** | IGLV3-27 | IPI00552852 | Q5NV91 | 3.6 | 0.9 | 3.6 | |  | |
| **Up-regulated in N** | | | | | | | | | |
| **Alpha-2-macroglobulin** | A2M | IPI00478003 | P01023 | 3356.6 | 2808.8 | 2802.3 | Ref [5] | | |
| **Angiotensinogen** | AGT | IPI00032220 | P01019 | 235.8 | 127.6 | 121.4 | Ref [5] | | |
| **Antithrombin-III** | SERPINC1 | IPI00032179 | P01008 | 747.5 | 341.0 | 587.6 | Ref [6] | | |
| **Apolipoprotein L1** | APOL1 | IPI00186903 | O14791 | 28.9 | 11.7 | 21.5 | Ref | | |
| **Coagulation factor V** | F5 | IPI00478809 | P12259 | 10.2 | 3.3 | 5.4 |  | | |
| **Complement C1q subcomponent subunit A** | C1QA | IPI00022392 | P02745 | 82.8 | 10.0 | 8.9 |  | | |
| **Complement C3** | C3 | IPI00783987 | P01024 | 3380.1 | 3341.6 | 2639.9 | Ref [4,5] | | |
| **Complement component C8 alpha chain** | C8A | IPI00414018 | P07357 | 103.2 | 65.4 | 84.3 |  | | |
| **Complement component C8 beta chain** | C8B | IPI00294395 | P07358 | 118.7 | 43.6 | 42.5 |  | | |
| **Extracellular matrix protein 1** | ECM1 | IPI00645849 | Q16610 | 1.4 | 0.0 | 0.4 |  | | |
| **Gelsolin** | GSN | IPI00026314 | P06396 | 101.3 | 47.5 | 67.2 | Ref [5,10] | | |
| **Hepatocyte growth factor activator** | HGFAC | IPI00029193 | Q04756 | 1.2 | 0.0 | 0.3 |  | | |
| **Ig gamma-2 chain C region** | IGHG2 | IPI00399007 | P01859 | 1195.8 | 987.5 | 1041.0 |  | | |
| **Ig gamma-4 chain C region** | IGHG4 | IPI00550640 | P01861 | 1297.6 | 1058.2 | 1091.1 |  | | |
| **Ig heavy chain V-III region TIL** |  | IPI00382478 | P01765 | 83.9 | 43.3 | 38.2 |  | | |
| **Ig heavy chain V-III region TUR** |  | IPI00382497 | P01779 | 77.4 | 39.0 | 34.9 |  | | |
| **Ig kappa chain V-I region Ni** |  | IPI00387106 | P01613 | 6.3 | 2.7 | 2.7 |  | | |
| **Ig kappa chain V-III region HAH** |  | IPI00030205 | P18135 | 218.8 | 141.4 | 90.8 |  | | |
| **Ig kappa chain V-III region NG9** |  | IPI00387116 | P01621 | 8.8 | 3.2 | 2.5 |  | | |
| **Ig mu heavy chain disease protein** |  | IPI00385264 | P04220 | 569.2 | 191.8 | 201.3 |  | | |
| **IGHD protein** | IGHD | IPI00418422 | P01880 | 28.1 | 11.0 | 9.9 |  | | |
|  |  | IPI00163446 |  |  |  |  |  | | |
| **IGHM protein** | IGHM | IPI00785207 | Q6GMX5 | 730.1 | 271.7 | 268.7 |  | | |
|  |  | IPI00748158 | Q86TT1 |  |  |  |  | | |
|  |  | IPI00549291 |  |  |  |  |  | | |
|  |  | IPI00477090 | Q8WUK1 |  |  |  |  | | |
| **Immunoglobulin heavy chain variable region** |  | IPI00745363 | Q0ZCH6 | 27.9 | 2.4 | 1.7 |  | | |
|  |  | IPI00783909 | Q0ZCH0 |  |  |  |  | | |
|  |  | IPI00783094 | Q0ZCG4 |  |  |  |  | | |
|  |  | IPI00477804 | Q0ZCJ6 |  |  |  |  | | |
| **Immunoglobulin lambda heavy chain** |  | IPI00784942 |  | 991.9 | 828.9 | 921.2 |  | | |
| **Immunoglobulin light chain variable region** |  | IPI00736325 |  | 6.6 | 1.3 | 0.1 |  | | |
| **Inter-alpha-trypsin inhibitor heavy chain H2** | ITIH2 | IPI00305461 | P19823 | 553.2 | 270.8 | 326.9 |  | | |
| **Isoform HMW of Kininogen-1** | KNG1 | IPI00032328 | P01042-1 | 217.6 | 125.9 | 165.1 |  | | |
| **Isoform LMW of Kininogen-1** | KNG1 | IPI00215894 | P01042-2 | 215.2 | 116.7 | 152.7 |  | | |
| **Kallistatin** | SERPINA4 | IPI00328609 | P29622 | 17.8 | 8.9 | 9.9 |  | | |
| **Myosin-reactive immunoglobulin kappa chain variable region** |  | IPI00384402 | Q9UL86 | 8.2 | 2.8 | 2.5 |  | | |
| **Plasminogen** | PLG | IPI00019580 | P00747 | 370.3 | 247.6 | 279.3 | Ref [5] | | |
| **Platelet basic protein** | PPBP | IPI00022445 | P02775 | 5.6 | 0.8 | 0.5 |  | | |
| **Platelet factor 4** | PF4 | IPI00022446 | P02776 | 16.3 | 5.8 | 3.1 |  | | |
| **Platelet factor 4 variant** | PF4V1 | IPI00022295 | P10720 | 15.3 | 5.4 | 2.9 |  | | |
| **Protein AMBP** | AMBP | IPI00022426 | P02760 | 470.9 | 89.7 | 58.5 |  | | |
| **SERPINC1 protein** | SERPINC1 | IPI00165421 | Q7KYQ5 | 627.5 | 251.2 | 430.8 |  | | |
| **Similar to Ig gamma-2 chain C region** |  | IPI00736860 | Q49AS2 | 2029.7 | 1535.8 | 1788.8 |  | | |
| **Single-chain Fv (Fragment)** | scFv | IPI00748998 | Q65ZC9 | 47.4 | 1.6 | 0.4 |  | | |
| **Tetranectin** | CLEC3B | IPI00009028 | P05452 | 8.7 | 1.9 | 3.7 | Ref [5] | | |
| **V-type proton ATPase 116 kDa subunit a isoform 2** | ATP6V0A2 | IPI00000425 | Q9Y487 | 7.3 | 0.2 | 0.3 |  | | |
| **187 kDa protein** |  | IPI00164623 |  | 3817.0 | 3751.2 | 2839.5 |  | | |

^a^ Averaged PSMs (peptide hits) in Normal (N), AD and SCC group.

^b^ Raw PSMs identified in A549 and H1703 cells and conditioned media.

**Reference**

1. Patz EF, Jr., Campa MJ, Gottlin EB, Kusmartseva I, Guan XR, et al. (2007) Panel of serum biomarkers for the diagnosis of lung cancer. J Clin Oncol 25: 5578-5583.

2. Chatterji B, Borlak J (2009) A 2-DE MALDI-TOF study to identify disease regulated serum proteins in lung cancer of c-myc transgenic mice. Proteomics 9: 1044-1056.

3. Ueda K, Katagiri T, Shimada T, Irie S, Sato TA, et al. (2007) Comparative profiling of serum glycoproteome by sequential purification of glycoproteins and 2-nitrobenzensulfenyl (NBS) stable isotope labeling: a new approach for the novel biomarker discovery for cancer. J Proteome Res 6: 3475-3483.

4. Heo SH, Lee SJ, Ryoo HM, Park JY, Cho JY (2007) Identification of putative serum glycoprotein biomarkers for human lung adenocarcinoma by multilectin affinity chromatography and LC-MS/MS. Proteomics 7: 4292-4302.

5. Okano T, Kondo T, Kakisaka T, Fujii K, Yamada M, et al. (2006) Plasma proteomics of lung cancer by a linkage of multi-dimensional liquid chromatography and two-dimensional difference gel electrophoresis. Proteomics 6: 3938-3948.

6. Dowling P, O'Driscoll L, Meleady P, Henry M, Roy S, et al. (2007) 2-D difference gel electrophoresis of the lung squamous cell carcinoma versus normal sera demonstrates consistent alterations in the levels of ten specific proteins. Electrophoresis 28: 4302-4310.

7. Chatterji B, Borlak J (2007) Serum proteomics of lung adenocarcinomas induced by targeted overexpression of c-raf in alveolar epithelium identifies candidate biomarkers. Proteomics 7: 3980-3991.

8. Howard BA, Wang MZ, Campa MJ, Corro C, Fitzgerald MC, et al. (2003) Identification and validation of a potential lung cancer serum biomarker detected by matrix-assisted laser desorption/ionization-time of flight spectra analysis. Proteomics 3: 1720-1724.

9. Maciel CM, Junqueira M, Paschoal ME, Kawamura MT, Duarte RL, et al. (2005) Differential proteomic serum pattern of low molecular weight proteins expressed by adenocarcinoma lung cancer patients. J Exp Ther Oncol 5: 31-38.

10. Fujii K, Nakano T, Kanazawa M, Akimoto S, Hirano T, et al. (2005) Clinical-scale high-throughput human plasma proteome analysis: lung adenocarcinoma. Proteomics 5: 1150-1159.
