# Supplementary material for: Integrative Proteomics and Tissue Microarray Profiling Indicate the Association between Overexpressed Serum Proteins and Non-Small Cell Lung Cancer
Source: PLoS One. 2012 Dec 19;7(12):e51748. doi: 10.1371/journal.pone.0051748 (PMC3526638; doi:10.1371/journal.pone.0051748)
Supplement: Table S4 — The final MRM transitions of A1BG and LRG1 with their optimized fragmentor and collision energy. a L and A represent [13C6] Leucine and [13C3] Alanine for heavy isotopic peptides respectively. b * means the best transition pairs for the absolute quantification of the protein. c Both Q1 and Q2 were set at Unit Resolution. (DOC) [file pone.0051748.s004.doc]

**Table S4. The final MRM transitions of A1BG and LRG1 with their optimized fragmentor and collision energy.**

| **Target Protein** | **Protein MW (Da)** | **Signature Peptides^a b^** | **light or heavy** | **Q1^c^** | **Q3^d^** | **Fragmentor (V)** | **Collision Energy (V)** |
| --- | --- | --- | --- | --- | --- | --- | --- |
| A1BG | 54,273 | HQFLLTGDTQGR | light | 458.2 | 734.3 | 132 | 13 |
|  |  | HQFLLTGDTQGR ***** | light | 458.2 | 633.3 | 132 | 13 |
|  |  | HQFLLTGDTQGR | light | 458.2 | 576.3 | 132 | 9 |
|  |  | HQFLLTGDTQGR | light | 458.2 | 360.2 | 132 | 9 |
|  |  | HQFL**L**TGDTQGR | heavy | 460.2 | 734.3 | 132 | 13 |
|  |  | HQFL**L**TGDTQGR ***** | heavy | 460.2 | 633.3 | 132 | 13 |
|  |  | HQFL**L**TGDTQGR | heavy | 460.2 | 576.3 | 132 | 9 |
|  |  | HQFL**L**TGDTQGR | heavy | 460.2 | 360.2 | 132 | 9 |
|  |  | SGLSTGWTQLSK | light | 632.8 | 920.5 | 116 | 21 |
|  |  | SGLSTGWTQLSK | light | 632.8 | 819.4 | 116 | 17 |
|  |  | SGLSTGWTQLSK | light | 632.8 | 762.4 | 116 | 17 |
|  |  | SGLSTGWTQLSK | light | 632.8 | 576.3 | 116 | 17 |
|  |  | SG**L**STGWTQLSK | heavy | 635.8 | 920.5 | 116 | 21 |
|  |  | SG**L**STGWTQLSK | heavy | 635.8 | 819.4 | 116 | 17 |
|  |  | SG**L**STGWTQLSK | heavy | 635.8 | 762.4 | 116 | 17 |
|  |  | SG**L**STGWTQLSK | heavy | 635.8 | 576.3 | 116 | 17 |
| LRG1 | 38,178 | DLLLPQPDLR ***** | light | 590.3 | 725.4 | 140 | 13 |
|  |  | DLLLPQPDLR | light | 590.3 | 288.2 | 140 | 33 |
|  |  | DLLLPQPDLR | light | 393.9 | 500.3 | 116 | 9 |
|  |  | DLL**L**PQPDLR ***** | heavy | 593.4 | 725.4 | 140 | 13 |
|  |  | DLL**L**PQPDLR | heavy | 593.4 | 288.2 | 140 | 33 |
|  |  | DLL**L**PQPDLR | heavy | 395.9 | 500.3 | 116 | 9 |
|  |  | ENQLEVLEVSWLHGLK | light | 632.0 | 840.5 | 148 | 25 |
|  |  | ENQLEVLEVSWLHGLK | light | 632.0 | 705.4 | 148 | 13 |
|  |  | ENQLEVLEVSWLHGLK | light | 632.0 | 640.9 | 148 | 13 |
|  |  | ENQLEVLEVSWLHGLK | light | 632.0 | 454.3 | 148 | 33 |
|  |  | ENQLEV**L**EVSWLHGLK | heavy | 634.0 | 840.5 | 148 | 25 |
|  |  | ENQLEV**L**EVSWLHGLK | heavy | 634.0 | 708.4 | 148 | 13 |
|  |  | ENQLEV**L**EVSWLHGLK | heavy | 634.0 | 643.9 | 148 | 13 |
|  |  | ENQLEV**L**EVSWLHGLK | heavy | 634.0 | 454.3 | 148 | 33 |

^a^ **L** and **A** represent [^13^C_6_] Leucine and [^13^C_3_] Alanine for heavy isotopic peptides respectively.

^b^ * means the best transition pairs for the absolute quantification of the protein.

^c^ Both Q1 and Q2 were set at Unit Resolution
