# Supplementary material for: Integrative Proteomics and Tissue Microarray Profiling Indicate the Association between Overexpressed Serum Proteins and Non-Small Cell Lung Cancer
Source: PLoS One. 2012 Dec 19;7(12):e51748. doi: 10.1371/journal.pone.0051748 (PMC3526638; doi:10.1371/journal.pone.0051748)
Supplement: Table S5 — The performance of the final MRM assay. Note that extremely low inter-assay CVs can be achieved due to the less experimental variations in analyzing non-depletion and non-fractionation sera. a L represents [13C6] Leucine and [13C3] Alanine for heavy isotopic peptides respectively. bDetermined as the lowest concentration touched with S/N≥3. cDefined as lowest concentration in the linearity curve (R>0.99) with CV<20%. dInter-assay CV was determined by a pooled cancer serum sample in 5 experimental replicates. (DOC) [file pone.0051748.s005.doc]

**Table S5. The performance of the final MRM assay.** Note that extremely low inter-assay CVs can be achieved due to the less experimental variations in analyzing non-depletion and non-fractionation sera.

| **Target Protein** | **Best Peptide^a^** | **Best Transition** | | **LOD^b^** | | **LOQ^c^** | | | **Inter-assay %CV^d^** |
| --- | --- | --- | --- | --- | --- | --- | --- | --- | --- |
|  |  | **Q1 (L/H)** | **Q3 (L/H)** | **fmol of peptide** | **μg/mL of protein** | **fmol of peptide** | | **μg/mL of protein** |  |
| A1BG | HQFL**L**TGDTQGR | 458.2/460.2 | 633.3/633.3 | 0.72 | 0.39 | 2.16 | 1.17 | | 2.23 |
| LRG1 | DLL**L**PQPDLR | 590.3/593.4 | 725.4/725.4 | 0.82 | 0.31 | 7.38 | 2.82 | | 1.90 |

^a^**L** represents [^13^C6] Leucine and [^13^C3] Alanine for heavy isotopic peptides respectively.

^b^Determined as the lowest concentration touched with S/N ≥3.

^c^Defined as lowest concentration in the linearity curve (R>0.99) with CV<20%.

^d^Inter-assay CV was determined by a pooled cancer serum sample in 5 experimental replicates.
