# Supplementary material for: Integrative Proteomics and Tissue Microarray Profiling Indicate the Association between Overexpressed Serum Proteins and Non-Small Cell Lung Cancer
Source: PLoS One. 2012 Dec 19;7(12):e51748. doi: 10.1371/journal.pone.0051748 (PMC3526638; doi:10.1371/journal.pone.0051748)
Supplement: Table S6 — The serum levels of A1BG and LRG1 in all the 100 samples measured by MRM assays. Normal, age-matched normal controls; AD: Adenocarcinoma; SCC: Squamous cell carcinoma. (DOC) [file pone.0051748.s006.doc]

**Table S6. The serum levels of A1BG and LRG1 in all the 100 samples measured by MRM assays.** Normal, age-matched normal controls; AD: Adenocarcinoma; SCC: Squamous cell carcinoma.

| **Sample Subset** | **Sex** | **Age** | **Smoking status** | **STAGE** | **A1BG (μg/mL)** | **LRG1 (μg/mL)** |
| --- | --- | --- | --- | --- | --- | --- |
| **AD** | Female | 49 | No | IIIa | 415.9704775 | 65.09627729 |
| **AD** | Male | 42 | Yes | IIIa | 415.3768461 | 58.8687233 |
| **AD** | Female | 42 | No | IIIa | 409.6386367 | 43.1025844 |
| **SCC** | Male | 70 | Yes | Ia | 357.3718057 | 48.22476487 |
| **SCC** | Female | 72 | No | II | 470.9754201 | 76.4617984 |
| **SCC** | Male | 61 | Yes | IIa | 258.6166492 | 80.92680517 |
| **AD** | Female | 74 | No | I | 402.2809745 | 126.3546935 |
| **SCC** | Male | 59 | Yes | I | 395.2141782 | 63.28399999 |
| **SCC** | Male | 55 | Yes | III | 427.0863172 | 131.6706046 |
| **SCC** | Male | 70 | Yes | IIIb | 216.2034841 | 56.19093325 |
| **SCC** | Male | 70 | Yes | IIIa | 351.6490432 | 39.35608153 |
| **AD** | Female | 63 | No | III | 349.7585648 | 110.6607907 |
| **AD** | Female | 69 | No | IV | 229.2834268 | 73.13618978 |
| **SCC** | Male | 74 | Yes | III | 371.1786785 | 111.2661036 |
| **AD** | Male | 53 | Yes | III | 515.1643716 | 67.72478374 |
| **AD** | Female | 52 | No | III | 398.8957323 | 59.75414239 |
| **SCC** | Male | 58 | Yes | I | 428.3782797 | 88.36697619 |
| **AD** | Male | 51 | Yes | I | 390.0813714 | 126.2091654 |
| **SCC** | Male | 71 | Yes | IIIa | 410.740285 | 230.4008218 |
| **AD** | Female | 50 | No | I | 540.6880603 | 41.3117703 |
| **AD** | Male | 64 | Yes | Ia | 338.4445249 | 34.04472154 |
| **SCC** | Female | 72 | No | II | 239.9973664 | 231.7574289 |
| **AD** | Female | 70 | No | III | 446.7863053 | 164.746336 |
| **SCC** | Male | 61 | Yes | III | 333.2349551 | 68.47376827 |
| **SCC** | Male | 74 | Yes | IIa | 439.9663713 | 141.9702704 |
| **AD** | Male | 64 | Yes | III | 269.2295133 | 136.7807582 |
| **SCC** | Male | 74 | No | I | 398.7763856 | 336.8006155 |
| **SCC** | Male | 62 | Yes | III | 229.2096016 | 199.1529093 |
| **AD** | Male | 68 | Yes | III | 331.912325 | 172.7107526 |
| **AD** | Male | 73 | Yes | I | 253.8215336 | 218.0519748 |
| **SCC** | Male | 63 | Yes | IIb | 354.2462561 | 104.9976511 |
| **SCC** | Male | 72 | Yes | IIb | 389.4541249 | 87.55745109 |
| **SCC** | Male | 64 | Yes | IIb | 288.5219292 | 106.1527543 |
| **SCC** | Male | 71 | Yes | IIb | 345.8595574 | 66.66765181 |
| **SCC** | Male | 61 | Yes | IIb | 371.9608306 | 63.33991769 |
| **SCC** | Male | 70 | Yes | IIb | 472.2429307 | 66.83886019 |
| **SCC** | Male | 63 | Yes | IIb | 171.8099799 | 25.45294404 |
| **SCC** | Male | 72 | Yes | IIb | 369.0721085 | 52.32848224 |
| **SCC** | Female | 72 | No | IIIa | 326.019647 | 85.05987573 |
| **SCC** | Female | 41 | No | IIIa | 363.9761241 | 84.20483378 |
| **SCC** | Female | 71 | No | IIIa | 395.8489266 | 84.21488355 |
| **SCC** | Female | 55 | No | IIIa | 326.5187212 | 73.14130222 |
| **SCC** | Female | 70 | No | IIIa | 461.002731 | 68.50601731 |
| **SCC** | Female | 43 | No | IIIa | 332.8667804 | 57.03056261 |
| **SCC** | Female | 72 | No | IIIa | 434.7120377 | 84.17590309 |
| **SCC** | Female | 56 | No | IIIa | 290.2228908 | 39.88552399 |
| **SCC** | Female | 59 | No | IIIa | 465.8056763 | 185.568907 |
| **SCC** | Female | 58 | No | IIIa | 371.9919369 | 72.59614088 |
| **SCC** | Male | 68 | No | IIIa | 359.004225 | 78.07957382 |
| **SCC** | Male | 67 | No | IIIa | 330.9176039 | 49.54433499 |
| **SCC** | Male | 67 | Yes | IIIa | 302.6299222 | 69.87789442 |
| **SCC** | Male | 68 | Yes | IIIa | 194.205186 | 29.62317449 |
| **SCC** | Male | 49 | Yes | IIIa | 358.740623 | 98.71309859 |
| **SCC** | Male | 50 | Yes | IIIa | 290.9174634 | 149.7405242 |
| **SCC** | Male | 64 | Yes | IIIa | 352.9999858 | 96.26740643 |
| **SCC** | Male | 55 | Yes | IIIa | 376.1009751 | 76.02722852 |
| **SCC** | Male | 60 | Yes | IIIa | 419.1663986 | 47.08608229 |
| **SCC** | Male | 66 | Yes | IIIa | 265.0106082 | 174.314566 |
| **SCC** | Male | 65 | Yes | IIIa | 396.5279973 | 74.67173752 |
| **SCC** | Male | 67 | Yes | IIIa | 472.9974355 | 65.28119432 |
| **SCC** | Male | 66 | Yes | IIIa | 305.2754808 | 86.63096492 |
| **SCC** | Male | 50 | Yes | IIIa | 495.3500829 | 66.79470988 |
| **SCC** | Male | 52 | Yes | IIIa | 515.8203107 | 57.4158783 |
| **SCC** | Male | 62 | Yes | IIIa | 246.2583045 | 39.3807633 |
| **SCC** | Male | 56 | Yes | IIIa | 552.6611611 | 70.73345227 |
| **SCC** | Male | 59 | Yes | IIIa | 178.1034674 | 37.95556124 |
| **SCC** | Male | 67 | Yes | IIIa | 417.4469604 | 79.06748309 |
| **SCC** | Male | 66 | Yes | IIIa | 354.3731863 | 40.07959993 |
| **SCC** | Male | 68 | Yes | IIIa | 245.1706493 | 35.68817 |
| **SCC** | Male | 67 | Yes | IIIa | 377.8647286 | 59.92317914 |
| **Normal** | Male | 59 | Yes |  | 203.4434342 | 43.81046787 |
| **Normal** | Male | 46 | Yes |  | 335.8765823 | 33.41894293 |
| **Normal** | Male | 61 | No |  | 450.8217557 | 38.80255408 |
| **Normal** | Female | 62 | No |  | 269.0195149 | 28.54865444 |
| **Normal** | Female | 45 | No |  | 290.304721 | 30.59364931 |
| **Normal** | Male | 59 | Yes |  | 264.7316248 | 54.26400407 |
| **Normal** | Female | 48 | No |  | 356.2107138 | 29.39487563 |
| **Normal** | Male | 45 | Yes |  | 326.7616232 | 55.07264613 |
| **Normal** | Male | 39 | No |  | 299.2201146 | 53.02598194 |
| **Normal** | Male | 55 | Yes |  | 265.9059974 | 38.68258962 |
| **Normal** | Female | 68 | No |  | 282.6905376 | 36.72991171 |
| **Normal** | Female | 65 | No |  | 289.6587756 | 47.94866566 |
| **Normal** | Male | 76 | Yes |  | 177.6306835 | 29.88082338 |
| **Normal** | Male | 43 | Yes |  | 271.2053945 | 56.94608923 |
| **Normal** | Female | 61 | No |  | 199.2779251 | 35.23732708 |
| **Normal** | Female | 58 | No |  | 215.5062931 | 38.97674383 |
| **Normal** | Female | 45 | No |  | 218.6533747 | 36.50512624 |
| **Normal** | Female | 37 | No |  | 271.6927487 | 32.0409745 |
| **Normal** | Female | 47 | No |  | 259.0156112 | 49.67393445 |
| **Normal** | Female | 72 | No |  | 259.6422084 | 46.06408051 |
| **Normal** | Male | 46 | No |  | 189.1543862 | 46.26717379 |
| **Normal** | Male | 61 | Yes |  | 296.2999879 | 56.74148188 |
| **Normal** | Male | 71 | Yes |  | 225.4576547 | 39.3531328 |
| **Normal** | Female | 62 | No |  | 249.094063 | 54.62538522 |
| **Normal** | Female | 67 | No |  | 294.8716741 | 30.7002306 |
| **Normal** | Female | 55 | No |  | 250.6816098 | 45.52093374 |
| **Normal** | Female | 53 | No |  | 282.3745644 | 66.06799009 |
| **Normal** | Male | 54 | Yes |  | 255.171405 | 37.65981928 |
| **Normal** | Male | 66 | Yes |  | 260.1569237 | 34.34700266 |
| **Normal** | Male | 44 | Yes |  | 240.2833077 | 55.65928009 |
