# Supplementary figures and images for: Integrative Proteomics and Tissue Microarray Profiling Indicate the Association between Overexpressed Serum Proteins and Non-Small Cell Lung Cancer
Source: PLoS One. 2012 Dec 19;7(12):e51748. doi: 10.1371/journal.pone.0051748 (PMC3526638; doi:10.1371/journal.pone.0051748)

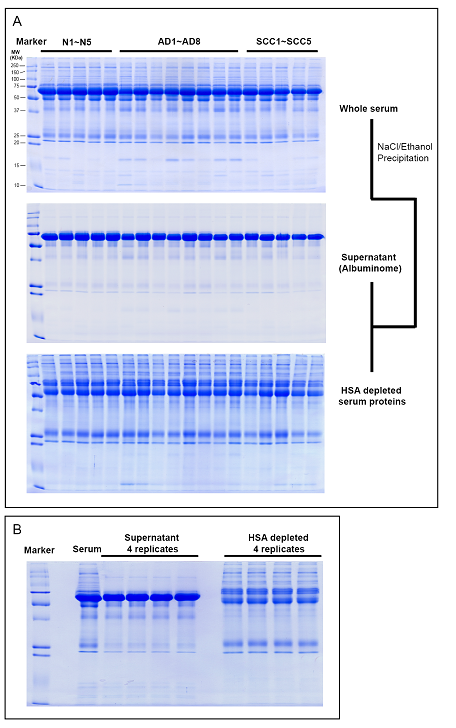

Supplement: Figure S1 — The albumin depletion for serum proteome. (A) Eighteen serum samples of the same starting volume were loaded for one dimensional electrophoresis. The resultant albuminome supernatant and HSA depleted pellet fractions decently showed quite analogous constitutes between individuals. (B) The streamlined protocol was also applied to one healthy serum and repeated for four times separately. Identical patterns were also observed from both supernatant and pellet fractions between these technical replicates. (TIF) [file pone.0051748.s007.tif]

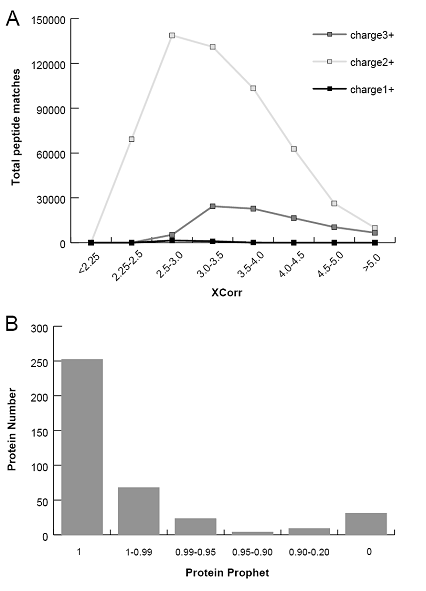

Supplement: Figure S2 — Xcorr distribution and peptide identification in serum proteome. (A) all of the spectra had a Xcorr higher than 2.25, and were dominated by identification of much higher scores of charge 2+ and charge 3+ ions. (B) Comparison between naive target-decoy protein FDR and Trans-Proteomic Pipeline (TPP) [26]. TPP was applied to all the raw spectra coming from one healthy serum. All the PSMs with PeptideProphet ≥0.75 were retained and assigned to proteins. Notably, 89.7% proteins in our identification result (by protein FDR) have a ProteinProphet ≥0.9. The proteins with a ProteinProphet of zero were all identified by multiple PSMs (33-25953 matches), and may be caused by the different peptide-protein group assignment priorities between TPP and Buildsummary. In contrast, if we retained the decoy tag in TPP, the final serum proteins with ProteinProphet ≥0.9 has a protein FDR equaled 10.7%, suggesting the fairly high confidences of our serum proteome. (TIF) [file pone.0051748.s008.tif]

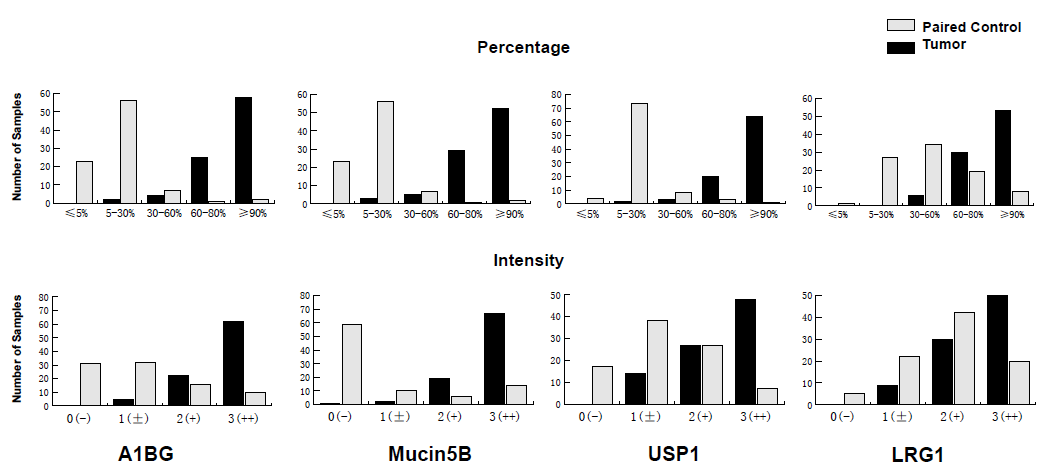

Supplement: Figure S3 — The distribution of the IHC staining intensity (I) and percentage (P) of positive stained cells. Both of them characterized most tumor sections with higher intensity (≥2) and more positive cells (>60%). (TIF) [file pone.0051748.s009.tif]

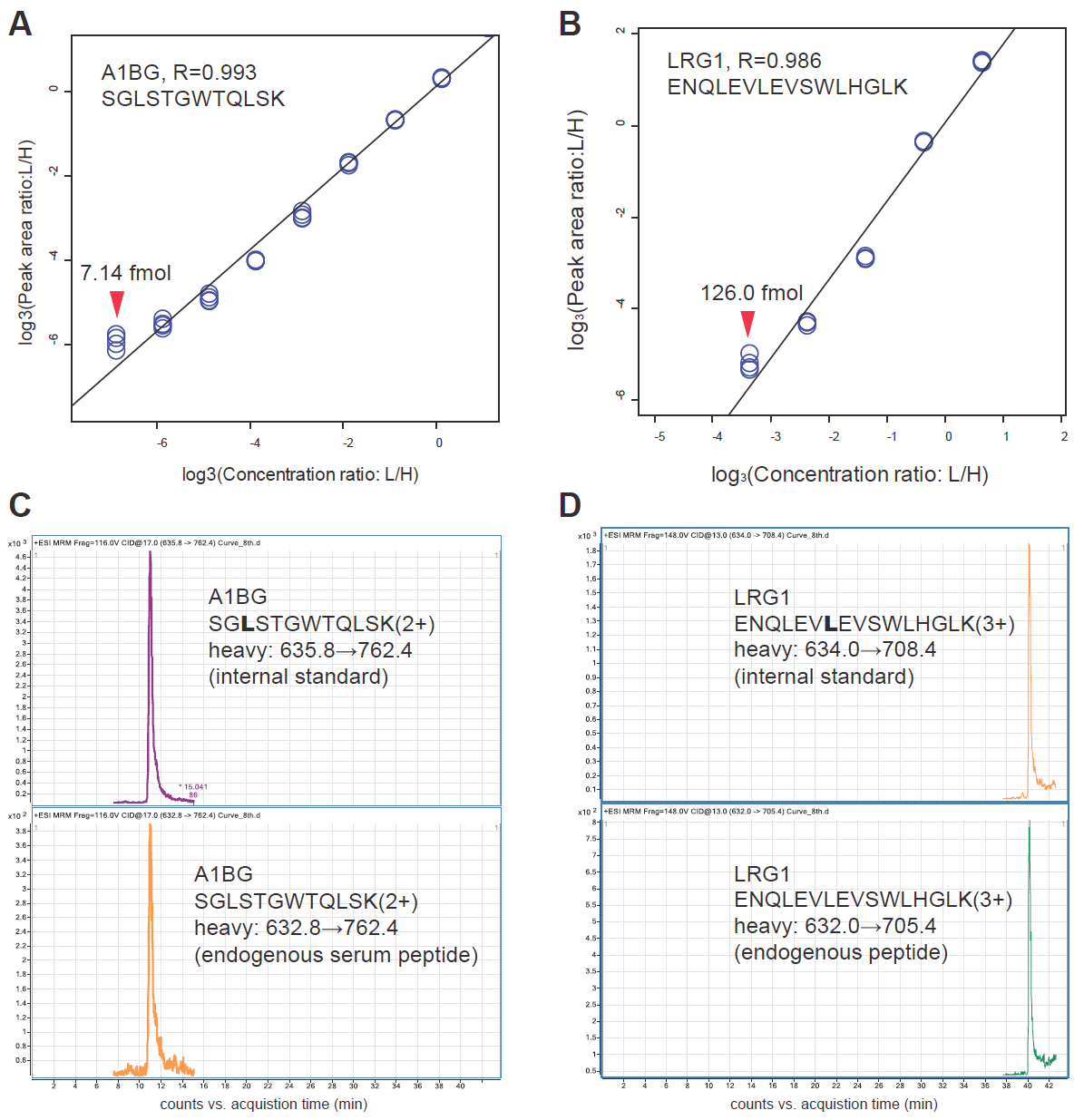

Supplement: Figure S4 — The MRM assays of the two less optimal reference peptides (compared to Figure 6 ) for A1BG and LRG1 measurements. MRM Intensities of two peptides both showed good or modest linear correlation with on-column abundance. The x-axis represents base-3 logarithm of ratios of spiked light and heavy isotopic peptides, with the y-axis corresponding to the observed peak area ratios in base-3 logarithmic scale. Red triangles suggest the limit of linear quantification (LOQ) of each peptide. (C–D) The chromatography peaks of the best transitions for two less-optimal peptides. L in bold indicates the pure, heavy [13C6] Leucine. (TIF) [file pone.0051748.s010.tif]
